# Symposia in undergraduate medical education: tailoring training in competencies to students’ needs

**DOI:** 10.1007/s40037-017-0379-4

**Published:** 2017-10-25

**Authors:** Karin Reefman, Hester E. M. Daelmans, Ursula M. H. Klumpers, Gerda Croiset

**Affiliations:** 1 VUmc School of Medical Sciences, Institute of Education and Training, Amsterdam, The Netherlands; 2 GGZ inGeest, Mental Health Institute, Amsterdam, The Netherlands; 3 LEARN! Research Institute for Learning and Education, Faculty of Psychology and Education, VU University Amsterdam, Amsterdam, The Netherlands

**Keywords:** Undergraduate medical education, Symposia, Competencies, Professional development

## Abstract

**Introduction:**

In mastering competencies, it is a challenge to create training sessions which acknowledge individual students’ needs and are logistically feasible in the medical master’s program.

**Methods:**

Symposia were implemented in the medical master’s program to provide knowledge and training of skills in a number of topics, providing a positive contribution to students’ competencies and personal development. Each symposium contained a morning and afternoon program, structured around medical and societal themes addressing various competencies and covering current national and international events. Alternating interactive teaching methods were used. Students were asked to rate each daypart program on a 5-point Likert scale in terms of both teaching methods and content, and to comment on the best aspects of the symposium as well as areas for improvement. Scores higher than 3.5 were interpreted as a predominantly favourable outcome.

**Results:**

In 2016, 10 symposia were organized with an average of 108 attendees and a response rate of 63% (1,366 completed questionnaires). Mean overall scores on ‘teaching methods’ and ‘usefulness for professional development’ were 3.8 and 3.7, respectively. The overall results corresponded with a high level of student appreciation.

**Conclusion:**

Symposia offer a podium for training students in subject matter and competencies that is greatly appreciated. Using alternating interactive teaching methods, symposia are structured around medical and societal themes and adjusted to the latest developments and current events in healthcare. By allowing students to select the symposia they would like to participate in, a tailor-made medical master’s program in competencies is created.

## Introduction

The majority of the competencies of the seven CanMEDS roles [1] are assimilated and trained in the authentic context of the physician during clerkships. In addition, most medical schools offer their medical students scheduled training sessions to explicitly focus on competencies. A crucial factor in this context is that these training sessions should be implemented across all disciplines as there are no competencies related to only one discipline [2]. Although the importance of training competencies across all disciplines is clear, creating education in competencies is a challenge [3]. The timing of clerkships (rotation-to-rotation, year-to-year) across the curriculum creates a logistical problem.

In designing a new way to train competencies in the master’s program, several requirements were drafted. First, each student is an individual with different needs and expectations [4]. Especially in terms of competencies, there are large differences between students. Therefore it is too rigid to require all students to participate in exactly the same training sessions; that does not acknowledge their individual needs. It is important to allow students to decide what the focal points should be in their individual education. It was therefore considered necessary to offer choices in the content of the medical curriculum. Additionally, providing choices in learning supports autonomy, which in turn may enhance autonomous motivation [5]. Second, there was a desire for education to be tailored to the latest developments in healthcare to retain an up-to-date program with a possibility to adjust to current national and international events dominating the news and social media (Papers, Journals, Twitter etc.). Experience has shown that this benefits the attractiveness of education and engagement of students. Third, the Royal College of Physicians and Surgeons of Canada created a new role in the CanMEDs in 2015, the role of leader [6]. This role was labelled as an important item for future training.

To teach and train students in integrated competencies in a logistically feasible way, symposia were implemented throughout the medical master’s curriculum. Symposia seem to fit both the moment and target audience, as medical master’s students will soon graduate and participate in postgraduate symposia in a context of lifelong learning.

## Methods

### Design and delivery

The 3‑year competency-based medical master’s program of VU University Medical Center hosts 360 students per year and was revised in 2015, allowing new educational elements such as symposia to be implemented. The goal of the symposia is to provide knowledge and training of skills in a number of topics, thereby providing a positive contribution in students’ competencies and personal development. In each of the three years of the medical master’s program, four symposia are organized. Students are required to attend at least two symposia every year. Each symposium contains two themes in a morning and an afternoon program, resulting in a total of 24 different programs per year.

Each symposium is structured around medical and societal themes addressing the various competencies. The majority of these themes are prescheduled in a calendar. Nonetheless, the content is modified each year based on evaluation outcomes and adjusted in response to new developments in healthcare. Examples of recurring themes are ‘Ethics’, ‘Patient safety: Team resource management’, ‘e-Health: Being a physician in times of social media and Google-patients’ and ‘Personal and medical leadership’, offering hands-on workshops such as ‘Balancing work and life’ and ‘Professional negotiation’. Additional items focus on current hot topics in national and international news, allowing the curriculum to be as up to date as possible. For example, in spring 2016, healthcare in relation to refugees in Europe was addressed in both a biomedical context and in terms of intercultural competencies. In addition, themes are tuned in with the stage of the master’s program. Accordingly, a ‘job interview training’ takes place in the third and final master’s year.

During the symposia, various activating and interactive teaching methods are used such as lectures, documentaries, workshops, simulation games and theatre. In training for patient safety, serious gaming is used as evidence suggests that this type of simulation can successfully promote the competencies of medical expert, communicator and collaborator [7].

Another teaching method is moral case deliberation, in which ethical dilemmas are addressed. In these sessions healthcare professionals, in this case students, present the moral questions that emerge in their clerkships during a structured dialogue. An ethicist facilitates the learning process by using various conversation methods to stimulate awareness and development of moral competencies [8].

### Evaluation

After each daypart program students were asked to complete a questionnaire. We collected the data for all organized symposia in 2016. For each daypart program, a total of 8 predefined statements (as shown in Tab. 1) were combined with content-specific statements concerning the educational value and quality of each specific program. Students were asked to indicate to which degree they ‘agreed’ or ‘disagreed’ with the statements on a 5-point Likert scale. The least favourable option (− − = strongly disagree/very bad) was assigned with a score of 1, the most favourable option (++ = strongly agree/very good) with a score of 5. Based on these scores, a mean score was calculated for each statement. The mean scores were interpreted as follows: scores lower than 3.5 corresponded with a predominantly unfavourable outcome and scores higher than 3.5 with a predominantly favourable outcome. Furthermore, the questionnaire asked students to comment on the best aspects of the symposium and areas for improvement.

**Table 1:** Evaluation of symposia: Mean ratings (SD) for first, second and third master’s year and total

Statements		Students 1^st^ master’s year (*n* = 416)	Students 2^nd^ master’s year (*n* = 140)	Students 3^rd^ master’s year (*n* = 810)	Total (*n* = 1,366)
		Mean (1–5) (SD)	Mean (1–5) (SD)	Mean (1–5) (SD)	Mean (1–5) (SD)
It is clear why this theme was included in the symposium		3.8 (0.8)	3.9 (0.6)	4.2 (0.7)	4.1 (0.7)
The theme addressed in the daypart program was meaningful		3.6 (0.9)	3.7 (0.7)	4.0 (0.8)	3.9 (0.8)
Content was aligned with knowledge		3.4 (0.9)	3.6 (0.8)	3.8 (0.9)	3.7 (0.9)
Applied teaching methods were suitable		3.6 (0.8)	3.9 (0.7)	3.9 (0.8)	3.8 (0.8)
Available time for addressing theme was adequate		3.9 (0.9)	3.9 (0.8)	3.8 (0.9)	3.8 (0.9)
Daypart program was interesting		3.5 (0.9)	3.7 (0.8)	3.9 (0.8)	3.8 (0.9)
Daypart program was useful for professional development		3.3 (1.0)	3.5 (0.8)	3.9 (0.9)	3.7 (1.0)
		*% score*	*% score*	*% score*	*% score*
How would you rate the overall level of the content of the daypart program?					
	Far too low	5.5%	3.2%	1.0%	2.6%
	Too low	25.5%	15.1%	11.1%	16.0%
	Just right	68.8%	80.2%	87.4%	80.8%
	Too high	–	1.6%	0.4%	0.4%
	far too high	0.5%	–	0.1%	0.2%

*SD* standard deviation

All student data were anonymous. Only pre-existing data, gathered for quality management procedures, were used. Therefore, student consent for use of the data was not required. The ethical review board of the Netherlands Association for Medical Education concluded that no further ethical review was necessary and approval was given to conduct the study (NVMO-ERB, file number 942).

## Results

In 2016, 10 symposia, consisting of 2 dayparts each, were organized. The number of attendees varied, with a mean number of 108 students participating, resulting in 2,168 distributed questionnaires. The mean response rate was 63%, corresponding with 1,366 completed questionnaires. Tab. 1 provides an overview of results for the first, second and third master’s year and the overall scores.

The mean overall scores for both ‘applied teaching methods were suitable’ and ‘daypart program was interesting’ were 3.8 and the mean overall score for ‘daypart program useful for professional development’ was 3.7, with lowest scores in the first master’s year and highest scores in the third for all three statements. The overall results correspond with a high level of student appreciation of the applied teaching methods and content of symposia; it was clear to students why various themes were embedded, the available time was considered adequate and the level of content was rated as ‘just right’ by 80.8% of the students.

Comments upon ‘best aspects’ most often related to the various interactive teaching methods; comments on ‘areas for improvement’ were mainly related to duration of specific components of the program, i. e. too long or too short.

## Discussion

Due to the introduction of a revised curriculum with less students at the start, the maximum number of students in a master’s year had not yet been reached when the first symposia took place. This resulted in lower numbers of attendees in early 2016. Later in the year, approximately 135 attendees per symposium was the standard. For the same reason, lower numbers were seen in the second master’s year compared with the first and third year. Also, in the second master’s year, fewer symposia were organized, resulting in a total of 10 symposia, instead of 12, in 2016.

An increase in appreciation was seen for virtually all statements as students progress through the curriculum. A possible explanation for this trend may be that as students get closer to graduation, they are more attracted to a type of education that is common and fitting for the postgraduate situation, i. e. symposia. Another explanation may be that they become more involved in competence development as they progress through the curriculum. However, this is hypothetical and speculative, as the content of the programs also differs in themes, lecturers and trainers.

Organizing symposia is a labour-intensive affair and requires good teamwork. Providing potential speakers with guidelines containing key elements and tips for a successful program (e. g., presenting interactive teaching methods), using a timetable and ensuring a clear distribution of tasks between all the parties involved are helpful tools in allowing symposia to be efficacious and financially viable. Furthermore, symposia may well benefit from the academic standard common in medical schools, with easy access to highly qualified clinical researchers and staff, including nationally recognized keynote speakers on various issues.

## Conclusion

It is a challenge to create education in competencies that is logistically feasible during the master’s curriculum. Using alternating interactive teaching methods, symposia offer a podium for training students’ competencies in a way that is much appreciated by students. Allowing students to select symposia of interest meets the ambition to comply with individual students’ needs and expectations. In this way we have created a tailor-made medical master’s program in competencies. Therefore the use of symposia for education in competencies can be a valuable addition to every curriculum.

## References

[1] Frank JR, Danoff D (2007). The CanMEDS initiative: implementing an outcomes-based framework of physician competencies. Med Teach.

[2] Hawkins RE, Welcher CM, Holmboe ES (2015). Implementation of competency-based medical education: are we addressing the concerns and challenges?. Med Educ.

[3] Frank JR, Snell L, ten Cate O (2010). Competency-based medical education: theory to practice. Med Teach.

[4] Hur Y, Cho AR, Huh S, Kim S (2017). How do medical students differ in their interpersonal needs?. Bmc Med Educ.

[5] Kusurkar RA, Croiset G (2015). Autonomy support for autonomous motivation in medical education. Med Educ Online.

[6] Dath D, Chan M-K, Abbott C (2015). CanMEDS 2015: from manager to leader.

[7] Aggarwal R, Mytton OT, Derbrew M (2010). Training and simulation for patient safety. Qual Saf Health Care.

[8] Molewijk AC, Abma T, Stolper M (2008). Teaching ethics in the clinic. The theory and practice of moral case deliberation. J Med Ethics.

